# Supplementary material for: Adolescent Knowledge, Attitudes and Practices of Healthy Eating: Findings of Qualitative Interviews among Hong Kong Families
Source: Nutrients. 2022 Jul 12;14(14):2857. doi: 10.3390/nu14142857 (PMC9316895; doi:10.3390/nu14142857)
Supplement: Supplementary file 1 [file nutrients-14-02857-s001.zip › nutrients-1792028-supplementary.pdf]

Document S1: Interview guide

Introduction:

"Thank you for your contribution to research. This interview is to find out more about your perception and experience of adopting a healthy eating habit as a youth. The interview will last for about an hour. Please feel free to share your experience and opinions. There are no right or wrong answers. All the information you have shared during this interview will be kept anonymous. Your name will not be disclosed in any transcript or report."

"感謝你為研究付出。此訪問會了解您們對青年實行健康飲食的看法及經驗。本訪問需時約一小時。請自由分享您們對青年實行健康飲食之見解及經驗，回答是沒有對錯之分。你們分享的資料將會絕對保密；你們的姓名亦不會在任何抄本或報告公開。"

<u>*Opening question*</u>

1. "Can you please tell me about your eating habit in general?"
   "可以告訴我你平時的飲食習慣是怎樣嗎?"
   Probes: Family cooking/ meals
   在家做飯/ 和家人食飯

<u>*Key questions*</u>

2. "How much vegetables/ fruits/ high-sugar products/ high-salt foods do you usually eat? Can you give examples of these foods?"
   "你通常進食多少蔬果/ 高糖東西/ 高鹽東西? 可以提供這些食物的例子嗎?"
   Sub-question:
   - "Why do you have/ not have snacking behavior?" *(i.e. any food consumed in between normal meals)*
     "你為什麼進食/ 不進食零食?" *(指任何在正餐之間進食的食物)*

   Probes: Non-green vegetables/ sugary beverages/ desserts/ salty snacks/ processed foods
   非綠色蔬菜/ 含糖飲品/ 甜品/ 咸零食/ 加工食品
3. "Do you know the guidelines for a healthy diet? Can you give examples on the guidelines for different food groups?"
   "你知道一些健康飲食的建議嗎? 可以提供不同食物的建議嗎?"
   Sub-questions:
   - "How do you tell whether the food is high in sugar/ salt?"
     "你怎樣知道食物是否高糖/ 高鹽?"

- “What benefits can be gained by following these guidelines?”
  “按照這些建議進食有甚麼好處?”
- “How did you receive the information?”
  “你從哪裏獲得這些資訊?”

Probes: Food pyramid/ 2 plus 3 A Day/ EatSmart Restaurant/ Reduction of Dietary Sodium and Sugar
健康飲食金字塔/ 日日二加三/ 有營食肆/ 減鹽減糖運動

4. “How do you perceive these guidelines? Do you agree with them?
“你覺得這些建議如何? 你同意嗎?”
Sub-questions:
   - “Do you believe they are important to your health? Why?
     “你相信這些建議對你的健康重要嗎? 為什麼?”
   - “Are you willing to follow them? Why?”
     “你會否按照這些建議進食嗎? 為什麼?”
   - “How about your family/ peer?”
     “你的家人/ 朋友又覺得這些建議如何?”
5. “Are you able to follow these guidelines? Why can/ can’t you?”
“你覺得你能夠達到這些建議嗎? 為什麼能夠/ 不能夠?”
Sub-questions:
   - “What are the facilitators/ barriers for you to achieve them?”
     “有甚麼事情鼓勵/ 妨礙你做到?”
   - “How did you try to promote the facilitators/ resolve the barriers?”
     “你如何提升這些鼓勵/ 解決這些困難嗎?”

Probes: Family and community factors
家庭和社區因素

6. “What other factors influence your eating habit?”
“有甚麼其他因素會影響你的飲食習慣呢?”
Sub-question:
   - “Would you tell me how you handle these factors?”
     “可以告訴我你怎樣面對這些因素嗎?”

Notes: The questions will be first directed to the adolescent, and then the parent. Their answers will be compared to show the similarities and differences in their perceptions of the adolescent eating habit. This also serves the purpose of cross-validation.

Table S1. Illustrative quotes of key findings on adolescent KAP of healthy eating

KAP	Themes	Subthemes	Quotes
Knowledge	Dietary recommendations	Relative portions of food categories	"Understand to eat the grains the most, then vegetables; oil, salt and sugar the least, like the five levels of Food Pyramid." (A1, F, 18)
		Recommended daily servings or allowance	"Suggesting that, like two plus three daily, two servings of fruit and three servings of vegetables." (A20, F, 17)
		Underestimating the recommended servings	"2/3… not remember is it half or one bowl of vegetables, 1/3 bowl of meat… One apple [is recommended daily], half bowl for others like cherries and grapes." (A3, F, 14)
	Health outcomes of healthy eating	Observable short-term outcomes	"Easy to get fat [by eating snacks], and heaty." (A19, F, 12)
		Specific benefits of eating FV	"[Eating vegetables] helps growing taller, do not have pain over the body." (A8, M, 14)
		Long-term outcomes	"Eating excessive [snacks] may lead to high blood sugar, hypertension those [diseases]." (A10, F, 14)
	Nutrition content in food	Food sources of fat, salt and sugar	"Sweets [are high in sugar]... Fries and those deep-fried foods [are high in salt]... I think [consuming] more [salt] at lunch due to eating out." (A11, F, 13)
		Knowing food sources of nutrients	"Protein [for sports performance]… Yes, [consuming] mainly by chicken, or eggs." (A2, M, 16)
		Interpretation of nutrition label or claim	"…. be aware of sugar, a beverage with ten-something grams of sugar per 100 g to be high in sugar, and drinks like soymilk with 4 g per 100 g are low sugar, so will buy them." (A1, F, 18)
		Healthy snack options	"[We] have plant milk, sometimes… those peanuts, nuts… Seems like better to have a balanced diet, better to have nuts." (A20, F, 17)
	Access to healthy meals	Unhealthy cooking methods of restaurant and takeaway food	"Restaurants usually add more seasonings for the flavor. It is unreasonable to request for less salt, there is no such option. Basically, eating out is probably unhealthy, because unable to control the amount of seasonings." (A1, F, 18)
		Healthy cooking methods	"Will buy steamed rice [with meat on top] at night… because it feels like healthier." (A3, F, 14)
		Ways to identify healthier restaurant meals	"May be those family styles [are healthier] as I can control the food portion, unlike buffet." (A1, F, 18)
Attitudes	Outcome expectation of	Experience of negative outcomes from unhealthy eating habits	"I think it is very important, because have experienced eating something wrong, and felt unwell and stomachache; or may have fever, diarrhea and vomiting, so eating healthily is important." (A7, F, 14)

	Food preferences	Being only necessary for older adults, but not for young people	"The relationship [between food and my health] is… not much relevant… Yes, should be [related to age], something like immunity." (A5, M, 15)
		Taste preference for unhealthy food	"When eating out with classmates... [usually order] pizza, fried chicken and other things which are also with high fat… Of course [I] like, who do not?" (A2, M, 16)
		Perceived inferior taste of healthy food	"Will not [choose less sugar for beverages]… Do not like the light flavor." (A4, M, 14)
		Priority of health among other preferences	"[Drinking soymilk or milk because of] health and not getting fat… It is not exceptionally delicious, but [it is regarded as] nutritious and tasty." (A1, F, 18)<br>"[The children] care about whether they enjoy the taste, the satisfaction, but not the long-term [impact]." (P6, F, 42)
	Self-efficacy to adopt healthy eating	Assessing health by body shape	"There are many competitions, and you know what kind of body shape should be maintained, and you will gradually adapt [to healthy eating] which becomes a norm." (A2, M, 16)
		Strategies to eat healthily with friends or on their own	"My classmates from university or secondary school basically do not cook at dorm, but I insist to cook by myself. They have frequent gatherings, drinking at bars, which I do not join them to maintain good health." (A1, F, 18)
		Skills of food preparation	"[A barrier to serve fruit as snacks is] not knowing how to cut [or peel]." (A19, F, 12)
Practices	Grocery shopping for healthy food	Not a habit to read nutrition label or health claim	"[I] know how to read [nutrition label], but will not intentionally select… Indeed I know these [sugary drinks] are unhealthy, but it cannot help if [I] like drinking, I will keep on buying." (A18, M, 19)
		Reading nutrition label for healthy alternatives	"Have [read nutrition label]… [Read] sugar [content] when buying drinks… The lower the figure the better." (A9, F, 19)
		Accompanying parents for grocery shopping	"We usually go out to buy [Vitasoy], [son] also chooses low sugar... Not [choosing when he is alone], he knows my habit, like he sometimes drinks milk tea outside, I also request for low sugar low ice for bubble milk tea... He knows that." (P17, F, 52)
	Eating home-prepared meals	Parents preparing for the meals	"We cook rice for both meals [lunch and dinner]... simpler for lunch... [Son] will not comment, eat whatever is cooked." (P16, F, 48)
		Eating ready-to-eat or easy-to-cook food for breakfast	"I cook some noodles for them [as breakfast]... usually add eggs, ham, or fishballs, or wonton, dumplings [those easy-to-cook choices]." (P7, F, 51)

	Composing grains, vegetables and meat for lunch and/or dinner	"Usually there is a bowl of soup, a dish of pan-fried vegetables, and sometimes pan-fried... vegetables... like red and green bell peppers... with some sliced pork." (A18, M, 15)
	Unhealthy ingredients for self-cooking by adolescents	"[Son] will cook when feeling hungry… Sometimes cook instant noodles, sometimes boil an egg, or pan-fried an egg, a sausage." (P13, F, 48)
**Eating out in restaurants or takeaway food**	Eating out or takeaway food for lunch after school/ Availability of unhealthy eating out options	"If going to school, sometimes will eat with the classmates after school, going to the food court or McDonald's at Citygate." (A11, F, 13)
	Infrequent eating out with family or friends	"Very limited, usually go for buffet or hotpot, because the setting is capable for more people [to sit together]… once every 2-3 months on average." (A1, F, 18)
	Occasional buying unhealthy takeaway food for family meals	"Mostly order takeaway for lunch... More convenient, [child] loves to eat... [Usually order] pork, chicken.. Mainly eat meat... Those meal sets with drinks." (P8, F, 51)
**FV consumption**	Eating FV once a day	"Assigning 1 bowl of cooked vegetables to each person in one meal... Eating a bowl [of fruit] before dinner." (P21, F, 47)
	Parents preparing ready-to-eat fruit	"Eating one piece [of fruit] by each person? Cannot. Usually cut two big apples for the whole family, around 1/2 [apple per person]… More for watermelon." (P4, F, 56)
	Self-serving of fruit	"Usually eat [fruit] at around two to three o'clock [as snacks] or after dinner at night... I serve the fruit at the fridge by myself... usually eat those apples, guavas." (A11, F, 13)
	Eating vegetables at home for dinner	"Eat the bread alone [for breakfast]… because there will not be vegetables or tomato ready at home to serve with." (A3, F, 14)\
	Eating variety of FV	"Onions, carrots, bell peppers, pumpkins, those potatoes, try to eat in turn rather than eating Choy Sum in every meal. There are many types, many choices for vegetables, broccoli, and those gourds like white gourds... those bitter melons." (P25, M, 47)
**Snacking**	Infrequent buying and eating snacks	"Nearly no snacks [at home]… Seldom buy myself, very rare, will eat when occasionally want to, may eat no more than once a month." (A2, M, 16)

Habit of unhealthy snacking	"Not as many [as once per day], because sometimes mum may not buy those that I like drinking... Sometimes may not drink for a whole week, but it is also possible to drink 3 to 4 packs in one day." (A24, F, 13)
Eating snacks available at home	"[Eat] chips, biscuits and whatever... Ice cream... Have to eat snacks... Eat whenever want to... Available [at home]." (A18, M, 19)
Serving healthy food and homemade drinks as snacks	"There is fruit at my home at all times, will eat when hungry… or some milk, cheese that type [of dairy products] in the fridge." (A20, F, 17)
